# Dynamics of Face and Head Movement in Infants with and without Craniofacial Microsomia: An Automatic Approach

**DOI:** 10.1097/GOX.0000000000002081

**Published:** 2019-01-11

**Authors:** Zakia Hammal, Erin R. Wallace, Matthew L. Speltz, Carrie L. Heike, Craig B. Birgfeld, Jeffrey F. Cohn

**Affiliations:** From the * Robotics Institute, Carnegie Mellon University, Pittsburgh, Pa.; † Seattle Children’s Research Institute, Seattle, Wash.; ‡ University of Washington School of Medicine, Seattle, Wash.; § Seattle Children’s Hospital, Seattle, Wash.; ¶ Department of Psychology, University of Pittsburgh, Pittsburgh, Pa.

## Abstract

**Background:**

Craniofacial microsomia (CFM) is a congenital condition associated with malformations of the bone and soft tissue of the face and the facial nerves, all of which have the potential to impair facial expressiveness. We investigated whether CFM-related variation in expressiveness is evident as early as infancy.

**Methods:**

Participants were 113 ethnically diverse 13-month-old infants (n = 63 cases with CFM and n = 50 unaffected matched controls). They were observed in 2 emotion induction tasks designed to elicit positive and negative effects. Facial and head movement was automatically measured using a computer vision–based approach. Expressiveness was quantified as the displacement, velocity, and acceleration of 49 facial landmarks (eg, lip corners) and head pitch and yaw.

**Results:**

For both cases and controls, all measures of expressiveness strongly differed between tasks. Case–control differences were limited to infants with microtia plus mandibular hypoplasia and other associated CFM features, which were the most common phenotypes and were characterized by decreased expressiveness relative to control infants.

**Conclusions:**

Infants with microtia plus mandibular hypoplasia and those with other associated CFM phenotypes were less facially expressive than same-aged peers. Both phenotypes were associated with more severe involvement than microtia alone, suggesting that infants with more severe CFM begin to diverge in expressiveness from controls by age 13 months. Further research is needed to both replicate the current findings and elucidate their developmental implications.

## Abstract 2

Supplemental Digital Content is available in the text.

## INTRODUCTION

Craniofacial microsomia (CFM) is a complex congenital condition characterized by the underdevelopment of structures arising from the first and second pharyngeal arches.^1,2^ The phenotypic spectrum of CFM includes malformations of the orbits, ear, mandible, facial soft tissue, and facial nerve. Prevalence is approximately 1 in 3,500 live births^3^ and higher among boys and children from Hispanic and Native American families.^4^ Children with CFM have myriad health care needs that require multiple, cross-disciplinary evaluations and interventions, including surgeries that help restore facial form and function.^5^

Facial nerve palsy is one of the many anomalies associated with CFM^6^ and the extent of facial nerve involvement can be difficult to ascertain in infancy due to the patient’s inability to cooperate with a structured facial nerve examination. In older children, facial palsy is evaluated by asking children to imitate specific facial expressions that indicate the responsiveness of different branches of the facial nerve. However, with infants, clinicians must rely on informal observations of spontaneous facial expressions during clinic visits, a process that is potentially unreliable and may under-identify palsy, delaying the recognition of need for relevant treatments (eg, feeding/eating and speech interventions; reanimation surgery). Moreover, facial palsy in early childhood, either alone or in combination with scarring from surgical interventions, may undermine infants’ facial communications of emotions to caregivers. The caregiver’s recognition of and responsiveness to infant facial expressions is a critical developmental process that has been linked to children’s later attainment of emotion regulation and socialization skills.^7–9^ If the facial anomalies associated with CFM disrupt this process, psychosocial interventions could be used to minimize or prevent such problems.^10–12^ However, such an approach would require a novel, reliable method of assessing facial expressions in very young patients.

We are developing such a method, using standardized emotion induction tasks to elicit positive and negative facial expressions in 12- to 14-month-old infants with CFM (“cases”) and demographically similar infants without CFM (“controls”). In a previous study,^13^ we used human coders and a manual coding system (Facial Action Coding System for Infants and Young Children; Oster, 2016) to compare the facial responses of cases versus controls. Positive and negative emotion induction tasks successfully elicited the intended facial expressions, but overall expressiveness did not differ between the 2 groups of infants, and only modestly distinguished the phenotypic variants associated with CFM (eg, microtia with or without mandibular hypoplasia). These null findings may be related to the fact that this study relied upon manually coded observations of facial movement, and small and subtle, but important, movements may have been missed by the coders. These include the displacement, velocity, and acceleration of not only facial movements but also movements of the head as well. Head movement is potentially important because face and head movement are inseparable in emotion communication and are strongly coupled, together influencing how facial expressions of emotion are communicated and perceived.^14–16^ Second, this study relied upon manually coded observations of facial movement, and small and subtle, but important, movements may have been missed by the coders

In recent years, machine learning^17^ has enabled the use of automatic facial analysis (AFA), which tracks and quantifies facial and head movements directly from digital video data.^18^ AFA has made possible the reliable and efficient measurement of displacement, velocity, and acceleration of facial and head movement. In previous research, AFA has revealed strong associations between effects and the dynamics of facial and head movement.^19–21^

In the current study, we used AFA to investigate whether infants with and without CFM differ in the dynamics of facial and head movement during tasks designed to elicit positive and negative effects. Face and head movements were tracked from 2D video using a well-validated generic approach made possible by training the algorithm with high-resolution 3D face-scans.^22–24^ Expressiveness was operationalized as the displacement, velocity, and acceleration of automatically tracked facial landmarks and head pitch and yaw. We addressed 2 specific questions: Do infants with CFM differ from controls in terms of head and facial expressiveness? Are these group differences specific to phenotypic anomalies associated with CFM? Secondary analyses involving all participants examined the potential moderating influence of infant sex, ethnicity, and type of emotion induction (positive versus negative).

## METHODS

Participants were 113 ethnically diverse 13-month-old infants. Cases (n = 63) were recruited from hospital-based craniofacial centers at Children’s Hospital of Los-Angeles, Children’s Hospital of Philadelphia, Seattle Children’s Hospital, University of Illinois-Chicago, and University of North-Carolina-Chapel Hill. Inclusion criteria were (1) Have at least one of the CFM indicators developed by the Facial Asymmetry Collaborative for Interdisciplinary Analysis and Learning (FACIAL) network^25^ (microtia, anotia, facial asymmetry, preauricular or facial tag(s), epibulbar dermoid, macrostomia); (2) Be diagnosed by a regional craniofacial team; (3) Be between the ages of 12 and 24 months (or corrected age if born between 34 and 36 weeks’ gestation); (4) Have a legal guardian able to provide informed written consent and willing to comply with all study procedures; and (5) Be available for the duration of the study. Exclusion criteria were (1) Diagnosis of a known syndrome (eg, Townes-Brocks or Nager syndromes); (2) Presence of an abnormal karyotype or major medical or neurological condition (eg, cancer, cerebral palsy); (3) Premature birth (less than 34 weeks’ gestation); (4) Any circumstance that would preclude the family’s ability to participate fully in the research; (5) A sibling already participating in the Craniofacial Microsomia: Longitudinal Outcomes for Children Pre-Kindergarten (CLOCK) study; and (6) consenting parent unable to speak English or Spanish. Because the dynamics of facial movement can vary as children age, participants more than 15 months of age were excluded.

Controls (n = 50) were recruited through pediatric practices located near the hospitals from which cases were recruited. These sources were supplemented by flyers posted in pediatric practices and from available infant studies participant pools. Inclusion criteria were demographic characteristics that met frequency-matching criteria for the case cohort with respect to infant age and sex, socioeconomic status, and language spoken in the home (English or Spanish).

Exclusion criteria included (1) meeting one or more of the exclusionary criteria for cases; (2) diagnosis or history of any disorder, condition, or injury that would affect facial features; and age older than 15 months.

### Phenotypic Classification of Cases

We classified the participant’s phenotype with a case-by-case integration of standardized photographic ratings of facial features and data taken from a medical history interview and medical charts.^26^ The photographic protocol and classification method^27^ generated 3 phenotypic subgroups: Microtia only (absence of other CFM-related features; n = 16); Microtia plus mandibular hypoplasia (n = 38); and Other combinations of CFM- associated malformations (n = 9).

### Observational Tasks

Infants’ expressiveness was observed in response to 2 standardized emotion inductions, one intended to elicit positive affect and the other negative affect.^28^ For each task, infants were seated in a highchair in front of an experimenter and their mother to the side (**see figure, Supplemental Digital Content 1**, which displays observational procedure, http://links.lww.com/PRSGO/A954).

#### Positive Emotion Task (PosET)

The experimenter engaged the infant by blowing soap bubbles toward them and using her voice to build suspense and elicit surprise, amusement, or interest (**see figure, Supplemental Digital Content 2**, which displays examples of Negative Emotion Task (left) and Positive Emotion Task (right), http://links.lww.com/PRSGO/A955).

#### Negative Emotion Task (NegET)

The examiner first allowed the infant to play with an attractive toy and then covered the toy with a clear plastic bin just out of the infant’s reach, which typically elicited frustration, anger, or distress (**Supplemental Digital Content 2**). The NegET was terminated if the infant became too upset or at the mother’s request.

The 2 tasks (ie, PosET and NegET) were each repeated 1–3 times unless the infant became too distressed to continue. Both tasks were recorded using a Sony DXC190 compact-camera.

### Automatic Face Analysis (AFA)

A person-independent 3D face tracker (Zface, Jeni et al. 2016) was used to track the 3D coordinates of 49 fiduciary points and head pitch (ie, head nods) and yaw (ie, head turns) in each video frame [**see figure, Supplemental Digital Content 3**, which displays examples of AFA tracking results (head orientation pitch (green), yaw (blue), and roll (red), and the 49 fiducial points). http://links.lww.com/PRSGO/A956]. Tracking results were overlaid on the source videos and manually reviewed. Frames that could not be tracked or failed visual review were not analyzed.

#### Expressiveness Using Facial Movement Dynamics

The movement of the 49 detected 3D fiduciary points corresponds to the movement (without rigid head movements) of the corresponding facial features (ie, eyes, eyebrows, and mouth) and was used to measure expressiveness from facial movement. The displacement, velocity, and acceleration of each one of the 49 detected fiduciary points was computed. The root mean square (RMS) was then used to measure the magnitude of variation of the fiduciary points’ displacement, velocity, and acceleration, respectively. The RMSs of the fiduciary points displacement, fiduciary points velocity, and fiduciary points acceleration are referred to as facial displacement, velocity, and acceleration, respectively. Because the movements of individual points are highly correlated, principal components analysis was used to reduce the number of parameters. The first principal components of displacement, velocity, and acceleration accounted for 63%, 76%, and 75% of the respective variance and were used as measurements of facial expressiveness.

#### Expressiveness Using Head Movement Dynamics

Head angles in the horizontal (ie, pitch) and vertical (ie, yaw) directions were used. Head angles were converted into angular displacement by subtracting the overall mean head angle from each observed head angle within each valid segment (ie, consecutive valid frames). Angular velocity and angular acceleration for pitch and yaw were computed as the derivative of angular displacement and angular velocity, respectively. Similar to facial movement, the RMS was used to measure the magnitude of variation of the angular displacement, the angular velocity, and angular acceleration for pitch and yaw, respectively. RMSs of the angular displacement, angular velocity, and angular acceleration for pitch and yaw were used as measurement of head expressiveness.

### Statistical Analyses

#### Dependent Variables

Expressiveness was the primary outcome and was operationalized using the dynamics of facial and head movement during both the PosET and NegET. Facial expressiveness was operationalized as the displacement, velocity, and acceleration of the automatically tracked 49 landmarks. Head expressiveness was operationalized as the angular displacement, angular velocity, and angular acceleration of pitch and yaw, respectively.

#### Analyses

To confirm that the induction tasks elicited the targeted affects, general estimating equations using an independent correlation matrix was used to compare expressiveness between PosET and NegET after adjustment for case status, sex, and ethnicity (Hispanic or Latino versus non-Hispanic/non-Latino). Linear regression with robust standard error estimates was used to estimate differences in expressiveness between cases and controls, as well as differences across phenotype, after adjustment for sex and ethnicity.

Due to the exploratory nature of this research, *P* values were unadjusted for multiple comparisons and they did not serve as the sole basis for estimating the strength of the findings. Instead, we assessed the magnitude of observed effect sizes, their precision, and the consistency of these estimates across multiple measures and the 2 emotion induction tasks.^29^ Standardized mean difference effect sizes were calculated using a modification of Cohen’s d^30^ that divides the estimated mean difference by the RMS error of the model.

## RESULTS

Average age at the time of the assessment was 13.2 months (SD = 0.71; Table 1). Relative to controls, cases were more likely to be male, to be Hispanic or Latino, to receive Medicaid insurance, and to have received speech, language, or hearing services.

**Table 1.**
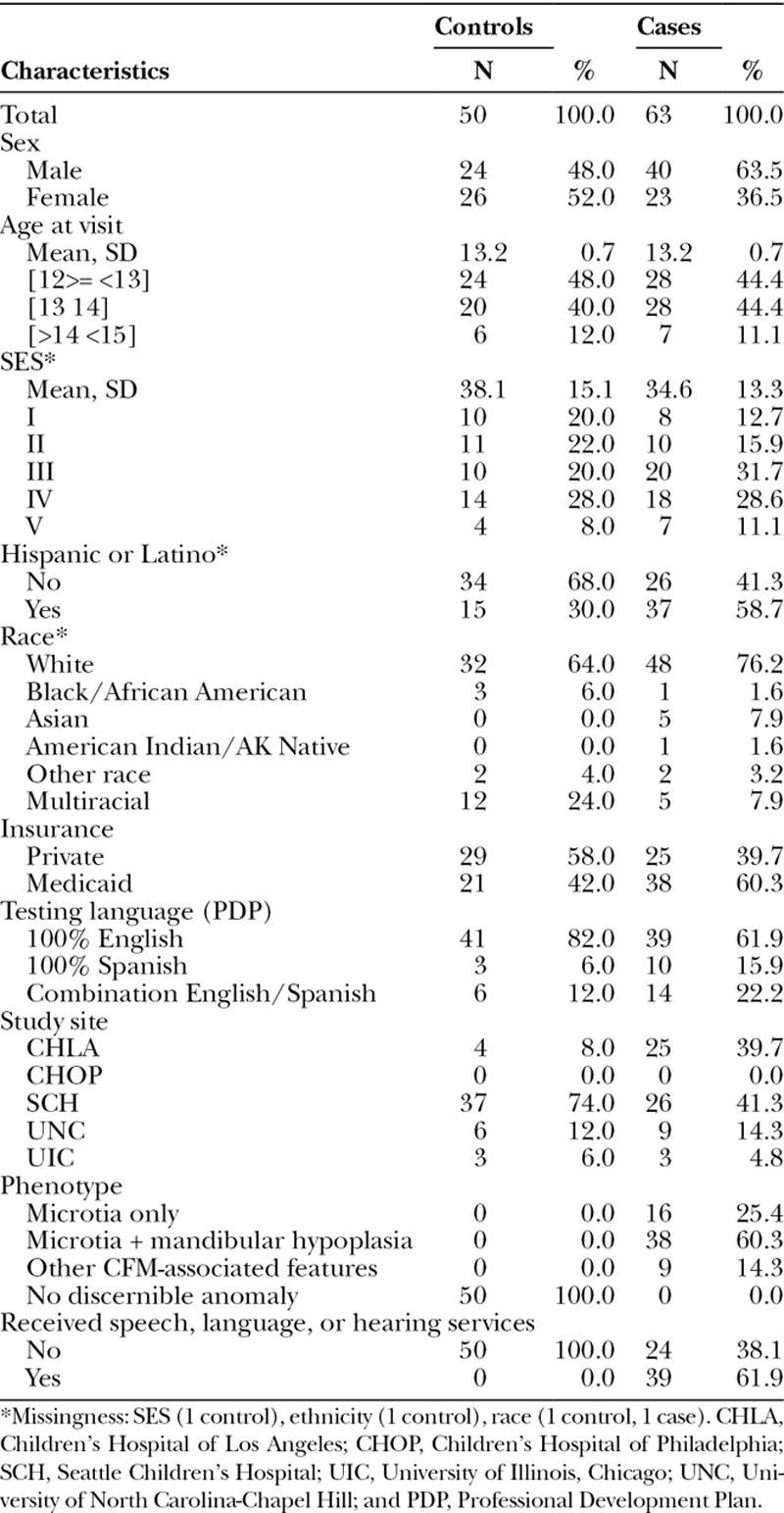
Characteristics of Infants with and without CFM

We observed strong differences in expressiveness between the positive and negative effect tasks across 19 out of 21 measures (*P* < 0.01). Head and face displacement, velocity, and acceleration were consistently greater during the NegET than PosET (Table 2).

**Table 2.**
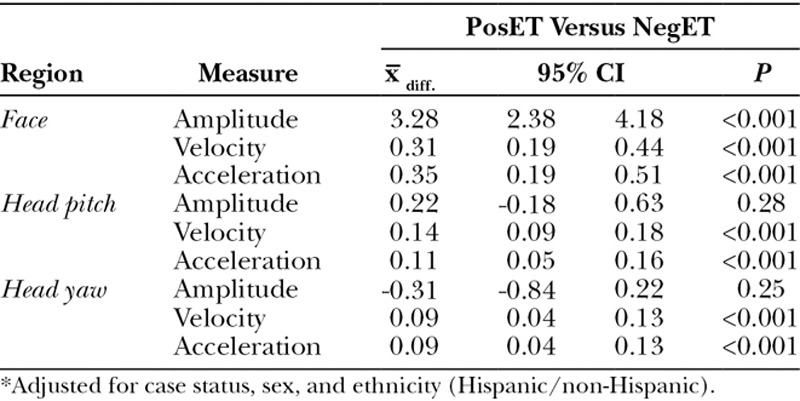
Adjusted Mean Difference in Expressiveness by Task*

### Case–Control Differences

After adjustment for sex and ethnicity, we observed little difference between cases and controls in holistic facial expressiveness and head movement (Table 3). The one significant finding (pitch displacement during the positive emotion task) was in the expected direction: less expressiveness in cases than controls.

**Table 3.**
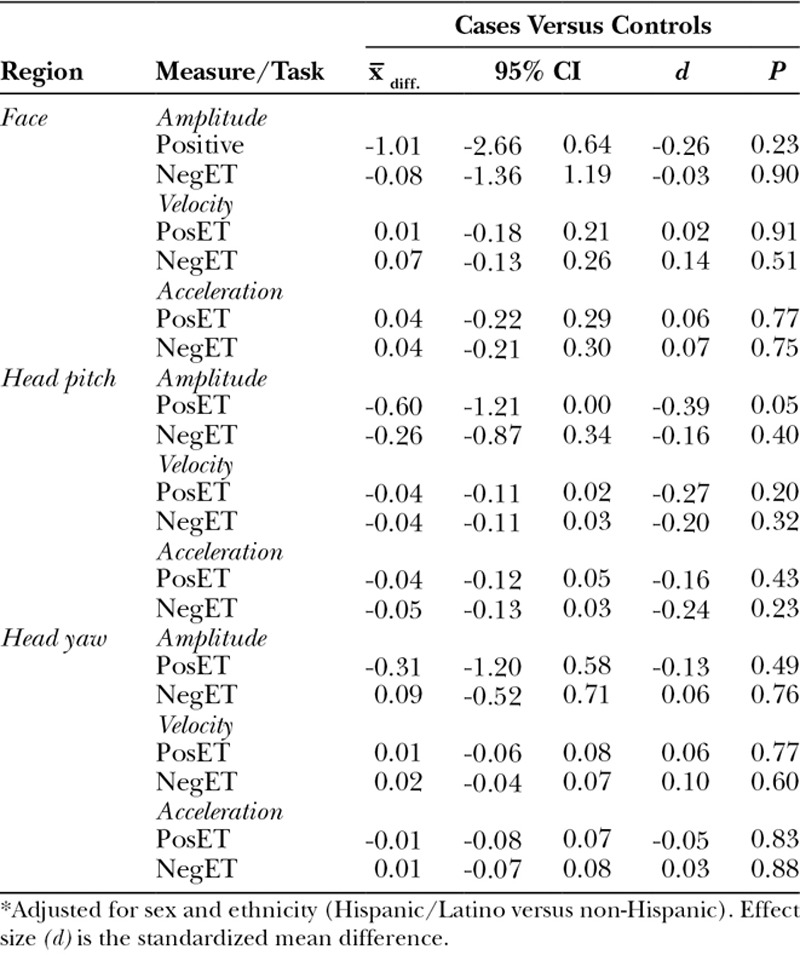
Adjusted Mean Difference in Expressiveness in Children with and without CFM*

### Analyses by Phenotype

Significant differences emerged between 2 of the 3 phenotypes and controls. For microtia with mandibular hypoplasia, face and head dynamics were significantly lower for cases than controls for 3 of 18 comparisons and 2 others were marginally significant (Table 4).

**Table 4.**
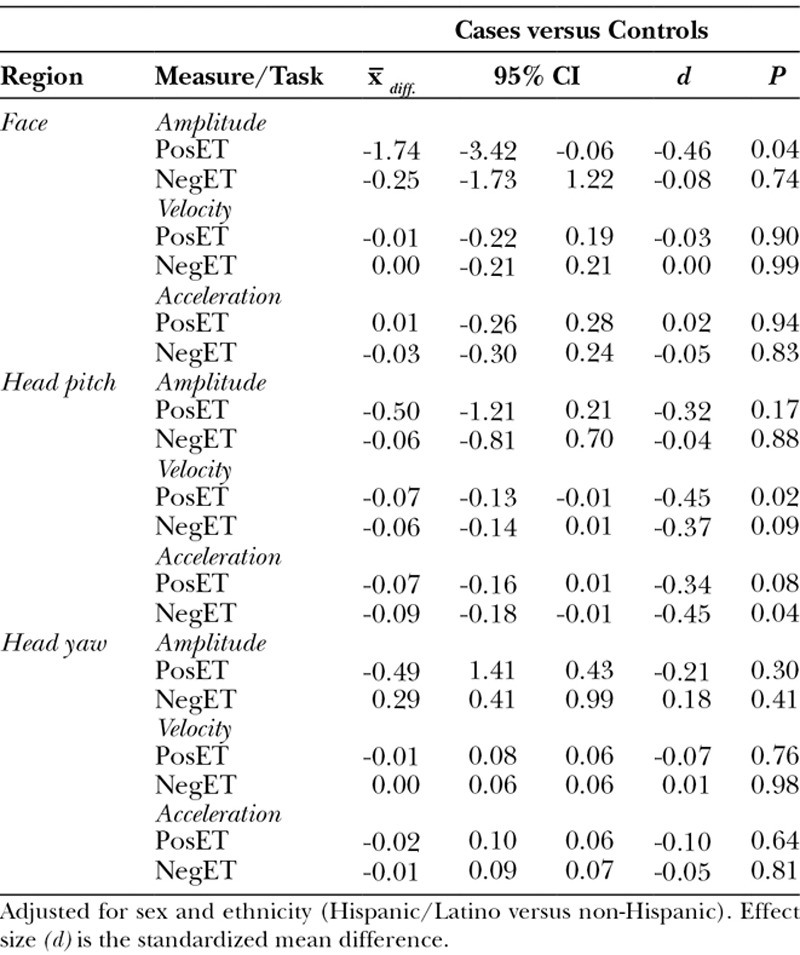
Adjusted Mean Difference in Expressiveness between Control Participants and Those with *Microtia Plus Mandibular Hypoplasia*

For other CFM-associated phenotypes, face and head dynamics were significantly lower for cases than controls for 2 of 18 comparisons and marginally significant for 2 others (Table 5). For microtia only, no differences were found between cases and controls.

**Table 5.**
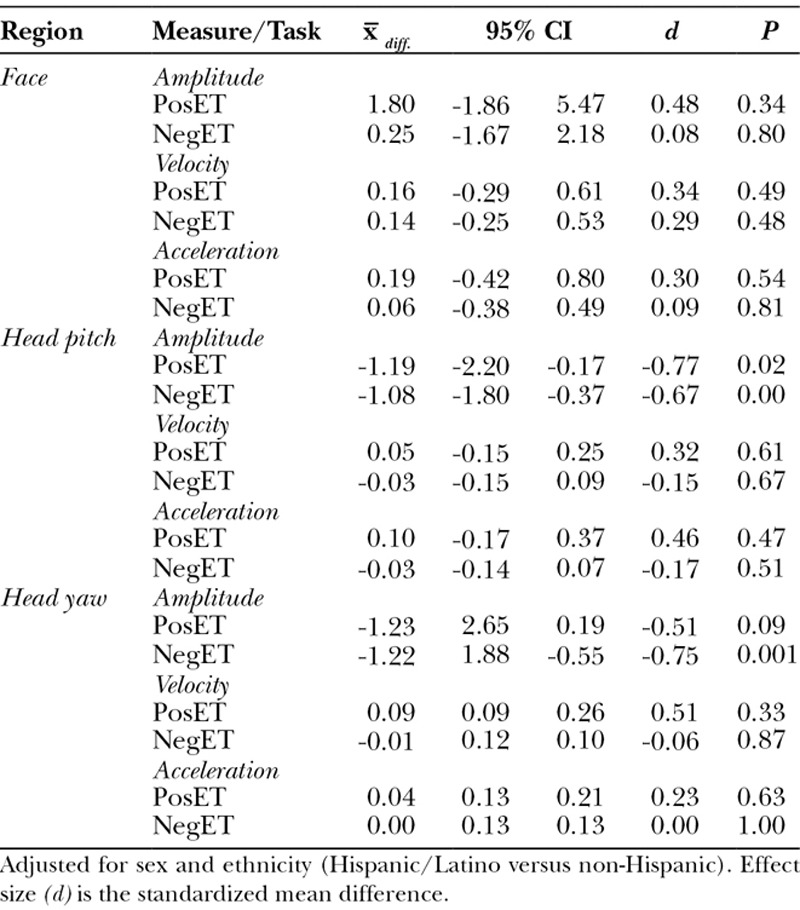
Adjusted Mean Difference in Expressiveness between Control Participants and Those with *Other CFM-associated Features*

**Table 6.**
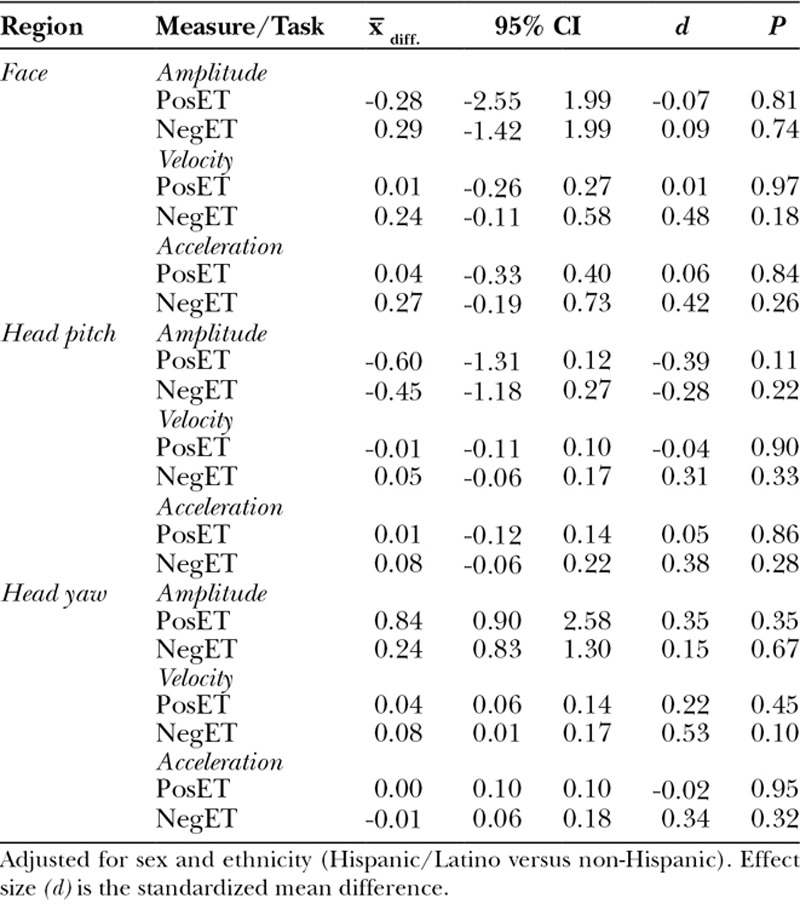
Adjusted Mean Difference in Expressiveness between Control Participants and Those with *Microtia Only*

### Secondary Analyses

After adjustment for case status and sex, Hispanic/Latino infants had lower levels of expressiveness in facial velocity and acceleration, pitch velocity and acceleration, and yaw velocity and acceleration. Effect sizes ranged from −0.7 to −0.1 with the strongest effects observed for the NegET (*P* values ranged from <0.001 to 0.71).

## DISCUSSION

The facial features associated with CFM increase risk for alterations in facial expressiveness and may lead to impairments in social and emotional development. We explored whether such effects emerge by the end of the first year of life and whether they vary among children with different phenotypic features in the broad category of CFM. To elicit individual differences in expressiveness, we used 2 well-validated emotion induction tasks that are known to provoke individual differences in affective reactivity. Expressiveness was objectively quantified using automated face analysis. Consistent with previous literature in both infants and adults, face and head movement displacement, velocity, and acceleration strongly differed between negative and positive tasks. Highly significant differences were found on 7 of 9 measures.

When we included all children with CFM, little evidence emerged for differences between infants with and without CFM. Of 18 comparisons between cases and controls, only one was statistically significant, which is about what one would expect by chance. However, when phenotype was considered, we observed more discernible differences between controls and 2 subgroups of cases. Cases with microtia plus mandibular hypoplasia and those with other-CFM associated features were less facially expressive than controls. During both positive and negative emotion tasks, the microtia plus mandibular dysplasia subgroup had lower displacement face movement and lower velocity and acceleration of head pitch (ie, head nods). The latter subgroup had lower displacement head pitch during both positive and negative tasks, lower displacement head yaw (ie, head turns) during the negative task, and marginally lower head yaw displacement in the positive task. No differences were found for the less severe, microtia only group. Nonetheless, we must acknowledge that adjustment for multiple comparisons (eg, Bonferroni correction) would require a *P* value of <0.001 to be considered statistically significant. However, as our analyses were exploratory in nature—using novel techniques to assess facial and head expressiveness—statistical significance is of less concern at this early stage of investigation. Furthermore, we are currently assessing facial expressiveness in the same cohort of children at approximately 3 years of age to see if we can replicate these patterns.

In comparison with the findings of Hammal et al.,^13^ the current findings more strongly suggest that CFM, and in particular individuals with microtia plus mandibular hypoplasia and other associated CFM features, is associated with a reduction in expressiveness as early as 13 months of age. Several factors may account for the increased sensitivity of our approach. First, the holistic measures were continuous, whereas Hammal et al^13^ used summed, binary measures (occurrence versus not occurrence), which emphasized the density of actions (ie, how many were occurring) rather than their intensity. Measurement of intensity was central to our approach and we sampled a far larger number of facial movements. In the prior study, they attended to 9 facial action units, whereas in the present study, we sampled 49 points across the face plus head pitch and yaw. Second, the temporal envelope of expressiveness was explicitly quantified in the current study (eg, velocity), whereas the action unit approach was insensitive to intensity change over time. Previous work in both infants and adults suggests that variation in displacement, velocity, and acceleration over time is strongly related to affect and interpersonal communication. Third, Hammal et al. used manual coding whereas our approach was fully automatic. Recent breakthroughs in computer vision and machine learning have made possible reliable automatic coding of action units that is consistent with experts’ manual coding.^31^ Although both human experts and algorithms now can code action units comparably, face and head movement dynamics can only be measured reliably by automatic algorithms. Reliable, automated measurement of face and head movement dynamics was necessary to further investigate expressiveness of infants with CFM.

An unexpected finding was that Hispanic infants were less expressive than non-Hispanic infants, regardless of cases status and phenotype. Previous research has found that Chinese infants are less expressive than Euro-American infants, with Japanese infants either comparable with Euro- American infants or between them and Chinese infants.^32^ Cross-cultural difference in expressiveness between Hispanic and non-Hispanic American infants have not previously been documented to our knowledge. For clinical purposes in evaluating expressiveness in Hispanic infants, it would be important to have use of separate norms for Hispanic and Euro-American non-Hispanic infants, as well as for East Asian infants.

Clinically, the present findings suggest that infants with only microtia have minimal risk for alterations in facial expressiveness. Elevated risk is suggested for infants with more severe phenotypic presentations of CFM. Increased monitoring and surgical or behavioral intervention may be indicated for these subgroups of patients.

Two limitations of the current study may be noted. One is that Hispanic infants were over-represented among cases relative to controls. As a consequence, ethnicity was included as a covariate in the analyses. Because ethnicity was related to expressiveness, controlling for ethnicity may have reduced sensitivity to detect CFM-related differences in expressiveness. We may have under-estimated CFM effects. The other limitation is the relatively small number of facial landmarks we quantified relative to what is possible. We sampled 49 landmarks. To provide denser sampling of facial movement, future work should consider using a larger number of landmarks.

## CONCLUSIONS

In this study, we have demonstrated the initial application of a novel, machine learning approach to the measurement of facial expressiveness in infants with a congenital condition that carries an elevated risk of facial palsy. Infants with CFM phenotypes beyond isolated microtia were less expressive than control infants. This finding, which requires replication, suggests that infants with more severe CFM begin to diverge in expressiveness from controls by 13 months of age. Longitudinal studies will be needed to learn whether these differences are stable or increase through early childhood, whether similar effects emerge for the less severe phenotype of microtia only and whether individual variation in facial expressiveness among infants with CFM predicts their psychosocial status in the preschool years.

## ACKNOWLEDGMENTS

The authors thank our colleagues Drs. Kathy Kapp-Simon (University of Illinois-Chicago), Amelia Drake (University of North Carolina-Chapel Hill), Alexis Johns (Children’s Hospital of Los Angeles), and Leanne Magee (Children’s Hospital of Philadelphia) at the participating craniofacial centers and the families who so generously volunteered their time to participate in this research.

## Supplementary Material
