# Supplementary figures and images for: Dynamics of Face and Head Movement in Infants with and without Craniofacial Microsomia: An Automatic Approach
Source: Plast Reconstr Surg Glob Open. 2019 Jan 11;7(1):e2081. doi: 10.1097/GOX.0000000000002081 (PMC6382247; doi:10.1097/GOX.0000000000002081)

Examiner

Mother

Baby

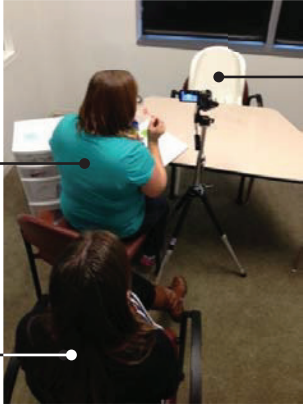

Supplement: Supplementary file 1 [file gox-7-e2081-s001.pdf]

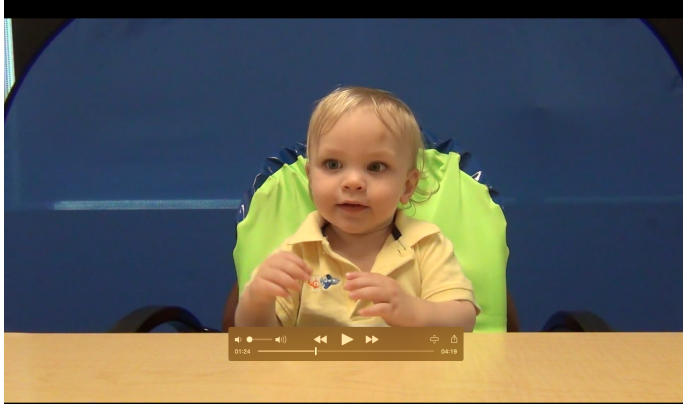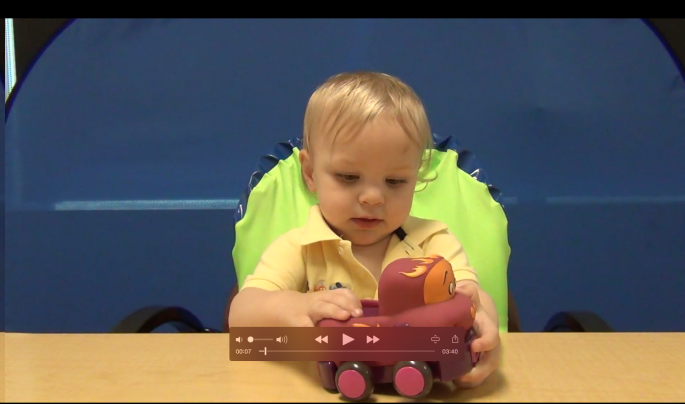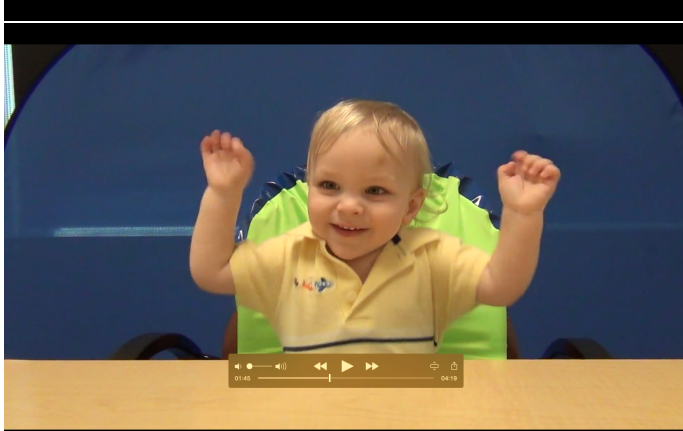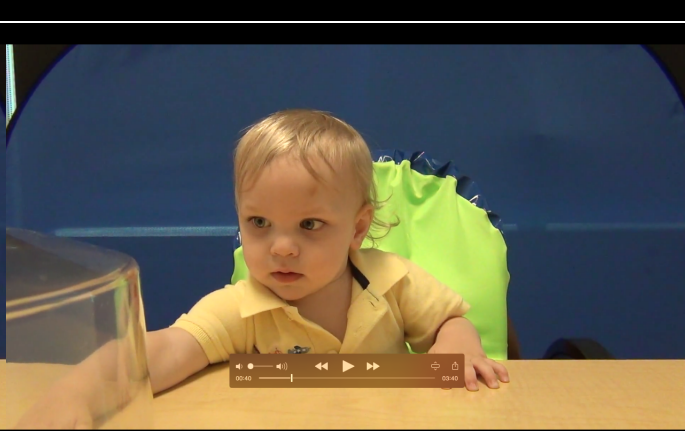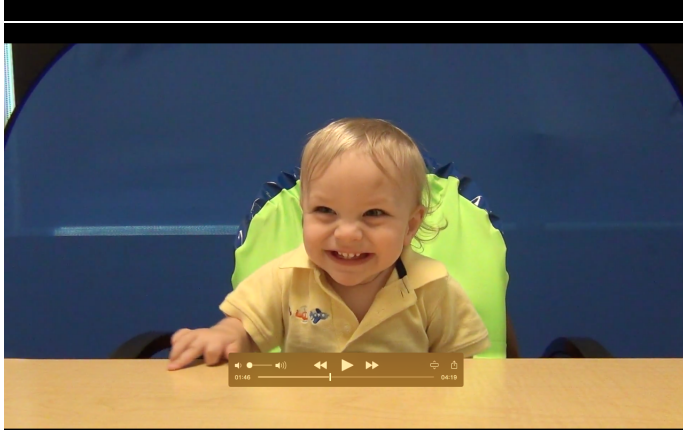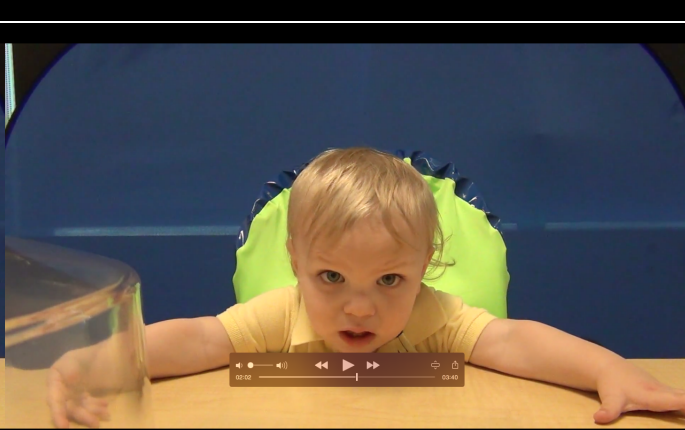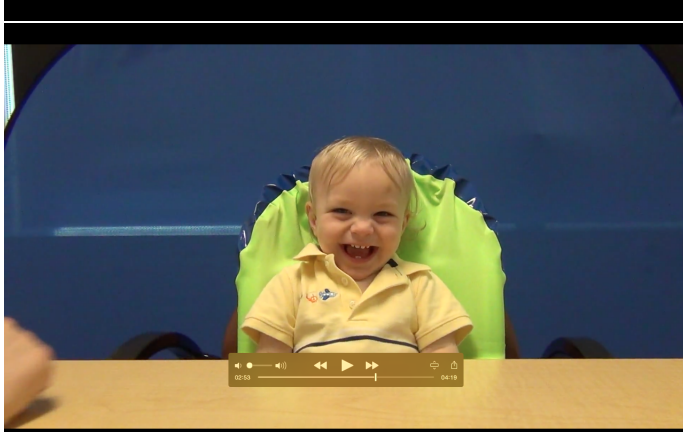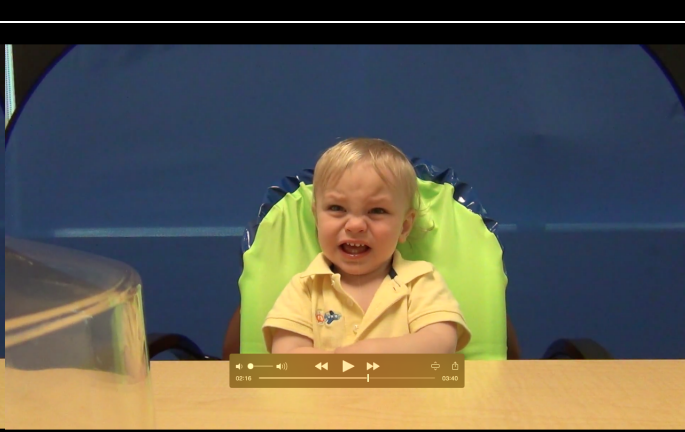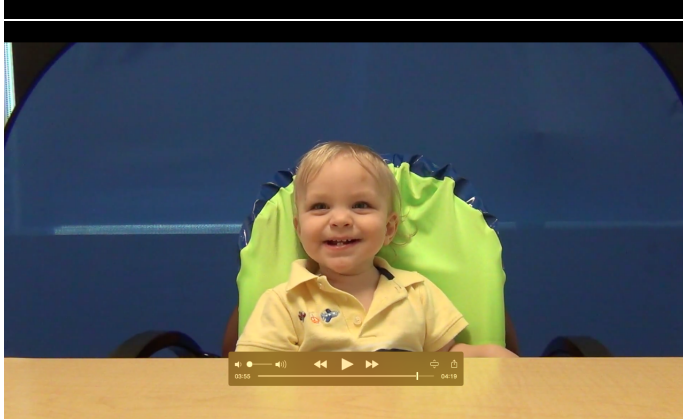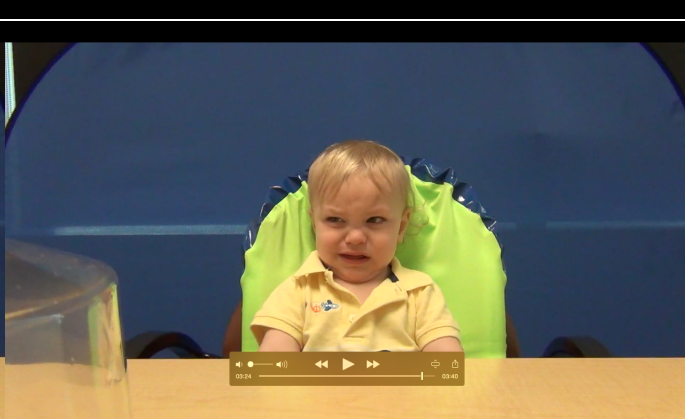

Supplement: Supplementary file 2 [file gox-7-e2081-s002.pdf]

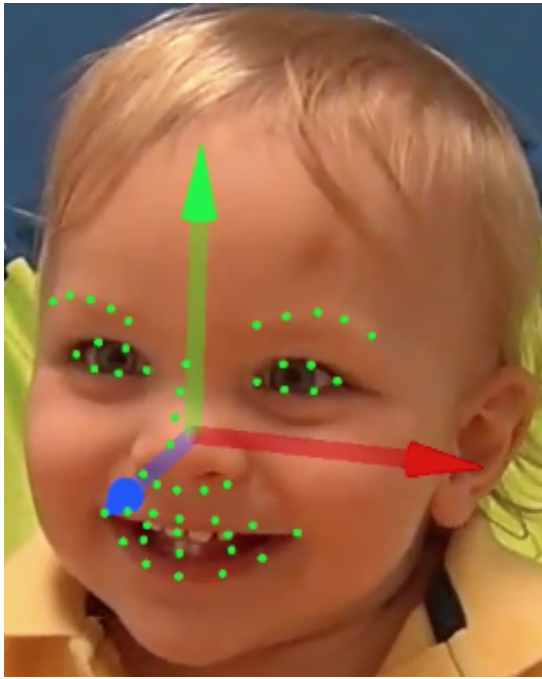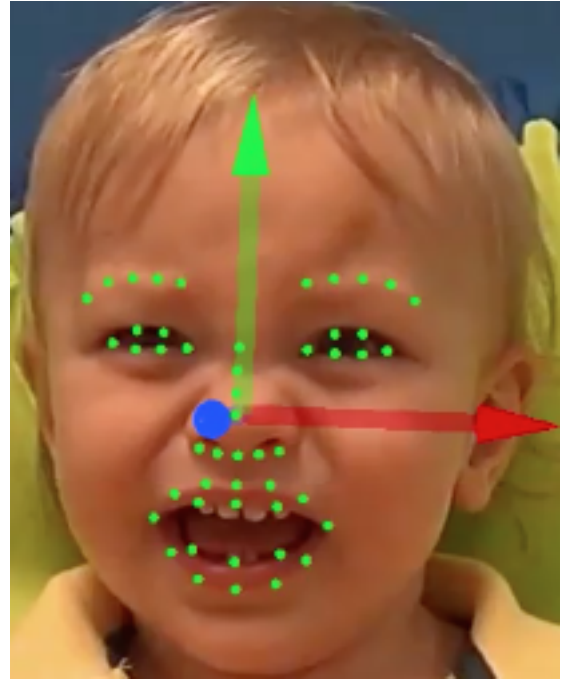

Supplement: Supplementary file 3 [file gox-7-e2081-s003.pdf]
